# Crystal structure of *trans*-bis­[2-(1*H*-benzotriazol-1-yl)acetato-κ*O*]bis­(ethano­lamine-κ^2^ *N*,*O*)copper(II)

**DOI:** 10.1107/S2056989019000744

**Published:** 2019-01-18

**Authors:** Guloy K. Alieva, Jamshid M. Ashurov, Shahnoza A. Kadirova, Bakhtiyar T. Ibragimov, Kasim A. Zakhidov

**Affiliations:** a Institute of General and Inorganic Chemistry of Uzbekistan Academy of Sciences, M.Ulugbek Str, 77a, Tashkent; b Institute of Bioorganic Chemistry, Academy of Sciences of Uzbekistan, M. Ulugbek Str 83, Tashkent; c Chemistry Department, National University of Uzbekistan, Tashkent; d Samarkand State University 140104, University blv. 15, Samarkand, Samarkand region, Uzbekistan

**Keywords:** copper(II), crystal structure, benzotriazol, mono­ethano­lamine, hydrogen bonding

## Abstract

The reaction of 2-(1*H*-benzotriazol-1-yl)acetic acid (HBTA; C_8_H_7_N_3_O_2_) and mono­ethano­lamine (MEA; C_2_H_7_NO) with CuCl_2_·2H_2_O resulted in the formation of the title complex, [Cu(C_8_H_6_N_3_O_2_)_2_(C_2_H_7_NO)_2_] or [Cu(BTA)_2_(MEA)_2_]. Its asymmetric unit comprises one BTA anion coordin­ating to the Cu^2+^ cation (site symmetry ) through the carboxyl O atom, and one MEA ligand chelating the metal cation by two heteroatoms (O and N). The equatorial Cu—O and Cu—N bond lengths are similar at 2.029 (1) and 1.980 (2) Å, respectively, while the length of the axial Cu—O bond is considerably greater [2.492 (2) Å], as is typical for Jahn–Teller-distorted systems. An intra­molecular hydrogen bond is present between the hy­droxy group of the MEA ligand and the non-coordinating O atom of the carboxyl­ate group. Inter­molecular hydrogen bonding involving the amino function of the MEA ligand and the carboxyl­ate group results in eight-membered rings with an *R*_2_^2^(8) graph-set motif. The mol­ecules are further linked by C—H⋯π inter­actions involving the triazole rings and methyl­ene groups of MEA, thus generating an overall three-dimensional supra­molecular framework.

## Abstract 2

The copper(II) atom shows a typical Jahn–Teller distorted [4 + 2] octa­hedral coordination sphere.

## Chemical context   

Recently, systematic studies of the structures and metal complex formation features of benzoic acid (Ibragimov *et al.*, 2016*a* ▸,*b* ▸) and benzotriazole derivatives have been carried out by our group. Benzotriazoles consist of nitro­gen-containing bicyclic ring systems and demonstrate many types of biological activities, such as anti­bacterial (Wan *et al.*, 2010 ▸; Suma *et al.*, 2012 ▸), anti­microbial (Nanjunda Swamy *et al.*, 2006 ▸; Singh *et al.*, 2009 ▸; Patel *et al.*, 2012 ▸; Ramachandran *et al.*, 2011 ▸), anti­fungal (Khabnadideh *et al.*, 2012 ▸; Rezaei *et al.*, 2009 ▸; Gaikwad *et al.*, 2012 ▸; Rakesh *et al.*, 2010 ▸), anti­cancer, anti-inflammatory, analgesic, anti­malarial and anti­tubercular (Kopańska (née Zastąpiło) *et al.*, 2004 ▸; Jamkhandi *et al.*, 2015 ▸). Functional groups such as carboxyl­ate, hydroxyl and pyridyl can be introduced to benzotriazole, increasing the coordination possibilities (Stoumpos *et al.*, 2008 ▸; Wang *et al.*, 2008*a* ▸,*b* ▸). The inter­action of metal ions with HBTA results in the formation of complexes in which it demonstrates monodentate (Ma *et al.*, 2015 ▸; Zeng *et al.*, 2012 ▸; Wang *et al.*, 2014*a* ▸) coordination. HBTA also can show bridging (Li *et al.*, 2016 ▸; Wang *et al.*, 2014*b* ▸) and *catena*-type (Wang *et al.*, 2011 ▸, 2014*b* ▸; Liu *et al.*, 2012 ▸) coordination modes. The inter­action of metal cations with MEA results in the formation of complexes in which MEA demonstrates monodentate (Hajji & Guerfel, 2016 ▸; Luo *et al.*, 2012 ▸; Ren *et al.*, 2014 ▸; Heinrich *et al.*, 2012 ▸; Guzei *et al.*, 2010*a* ▸) and bidentate (Ibragimov *et al.*, 2017 ▸; Seppälä *et al.*, 2013 ▸; Yeşilel *et al.*, 2012 ▸; Xue *et al.*, 2016 ▸; Ashurov *et al.*, 2015 ▸) coordination modes. In some complexes, MEA has bridging properties (Shahid *et al.*, 2015 ▸; Tudor *et al.*, 2013 ▸; Schwarz *et al.*, 2010 ▸; Maclaren *et al.*, 2012 ▸; Seppälä *et al.*, 2012 ▸). In addition, there are metal complexes in which MEA mol­ecules show non-coordinating behaviour (Wang *et al.*, 2013 ▸; Lemmerer & Billing, 2010 ▸; Calderone *et al.*, 2011 ▸; Yadav *et al.*, 2015 ▸; Sutradhar *et al.*, 2012 ▸; Liu *et al.*, 2011 ▸).
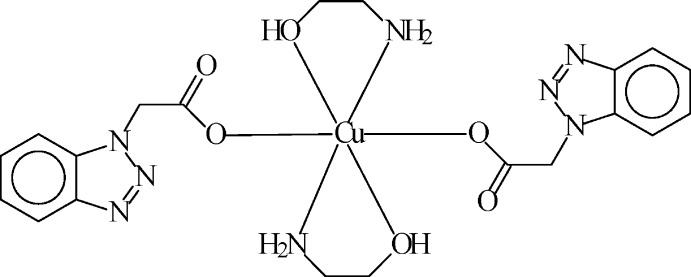


We have reported the synthesis of mixed-ligand complexes of Cu and Zn with MEA and α-naphthyl­acetic acid (NAA) and determined the structures of [Cu (NAA)_2_(MEA)_2_] and [Zn(NAA)_2_(MEA)_2_] (Ashurov *et al.*, 2015 ▸). A search in the Cambridge Structural Database (CSD Version 5.39, last update August 2018; Groom *et al.*, 2016 ▸) revealed that crystal structures have been reported for complexes of HBTA and MEA with many metal ions. However, no mixed-ligand metal complex including HBTA and MEA is documented in the CSD. Here, the synthesis and structure of the title compound, [Cu(BTA)_2_(MEA)_2_], (I), is described.

## Structural commentary   

**Figure 1:**
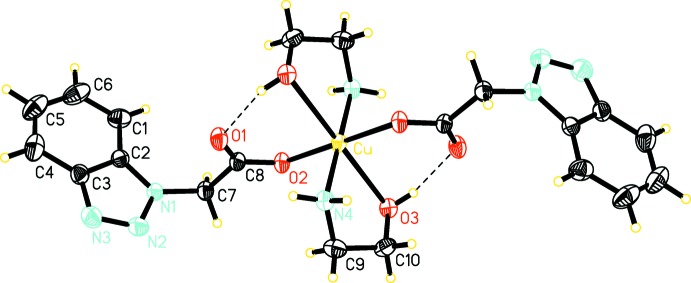
The mol­ecular structure of [Cu(MEA)_2_(BTA)_2_] with the atom-numbering scheme. Displacement ellipsoids are drawn at the 25% probability level.

The mol­ecular structure of *trans*-bis­(ethano­lamine-κ^2^ *N*,*O*)bis[2-(1*H*-benzotriazol-1-yl)acetato-κ*O*]copper(II), (I), is shown in Fig. 1 ▸ and consists of isolated [Cu(MEA)_2_(BTA)_2_] units. The Cu^2+^ cation is located on a center of inversion. Its coordination polyhedron is a distorted N_2_O_4_ octa­hedron formed by two oxygen atoms (O2) of the carb­oxy groups of symmetry-related BTA anions, by two nitro­gen atoms (N4) of two symmetry-related MEA ligands in the equatorial plane and by two O atoms (O3) of the same set of MEA ligands in the axial positions. The Cu—O2 and Cu—N4 bond lengths are 2.029 (1) and 1.980 (2) Å, respectively, whereas the length of the axial Cu—O3 bond is 2.492 (2) Å, typical for Jahn–Teller distortions. The MEA ligand is neutral and acts as a bidentate N- and O-donor ligand and forms CuNC_2_O five-membered chelate rings which have a twist conformation; the O3—C10—C9—N4 torsion angle is −60.3 (3)°. The planar benzotriazole ring system (N1–N3/C1–C6: r.m.s. deviation = 0.0064 Å) is co-planar with the methyl carbon atom C7 [deviation from the plane of 0.158 (2) Å], whereas the carboxyl­ate group is nearly normal to this plane [88.0 (2)°]. The difference of the C8—O(1,2) distances of the carboxyl­ate group (Δ = 0.036 Å) is due to the monodentate coordination, with the longer C—O distance involving the coordinating O2 atom.

The mol­ecular structure is stabilized by an intra­molecular O3—H3⋯O1 hydrogen bond between the OH group of the MEA ligand and the non-coordinating carboxyl­ate O atom (Fig. 1 ▸, Table 1 ▸).

## Supra­molecular features   

**Figure 2:**
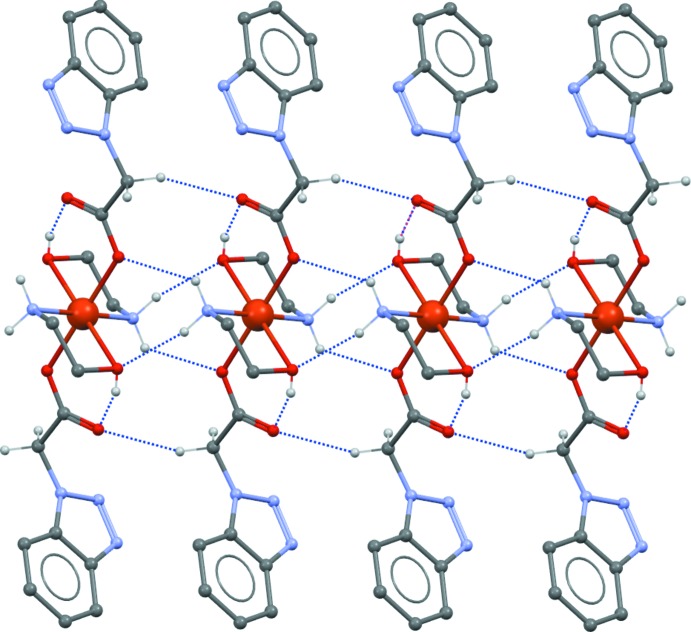
Chain structures formed by hydrogen bonds in the structure of (I). Hydrogen bonds are shown as dashed lines.

**Figure 3:**
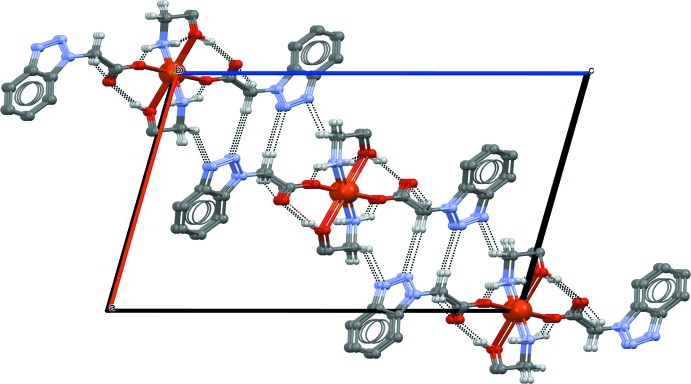
A partial view along the *b* axis of the crystal packing of compound (I). Inter­molecular hydrogen bonds are shown as dashed lines.

In the crystal structure of (I), mol­ecules are linked by C7—H7*A*⋯O1^iii^, N4—H4*A*⋯O2^ii^ and N4—H4*B*⋯O3^ii^ hydrogen bonds between the amino function and carboxyl­ate/hy­droxy O-atom acceptors (Table 1 ▸, Fig. 2 ▸), forming chains propagating parallel to [010]. Adjacent chains are linked by C9—H9*A*⋯N3^iv^ hydrogen bonds into a layered arrangement parallel to (10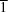) (Fig. 3 ▸). Additional C—H⋯π inter­actions between the triazole rings and methyl­ene groups of MEA (H⋯*Cg* = 2.88, C—H⋯*Cg* = 140°, 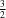 − *x*, 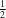 + *y*, 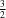 − *z*) generate a three-dimensional supra­molecular framework.

## Database survey   

There are thirty-one structures of coordination compounds that are derived from 2-(1*H*-benzotriazol-1-yl)acetic acid and different metal cations in the CSD (Version 5.39, last update August 2018; Groom *et al.*, 2016 ▸). The inter­action of metal ions with BTA results in the formation of complexes in which metals demonstrate monodentate [CUYGAG (Ma *et al.*, 2015 ▸), DUWQES (Zheng *et al.*, 2010 ▸), LAMYUV (Zeng *et al.*, 2012 ▸), TIVWOM (Wang *et al.*, 2014*b* ▸), TOBDUK (Hang & Ye, 2008 ▸)] and bridging [COHFOW (Ren *et al.*, 2013 ▸), DEZHIB (Zeng, 2013 ▸), GADVEP (Li *et al.*, 2016 ▸), TIVXAZ (Wang *et al.*, 2014*b* ▸)] coordination modes. BTA also can show *catena*-type structures [DEZHOH (Zeng, 2013 ▸), DUWQAO (Zheng *et al.*, 2010 ▸), DUWQIW (Zheng *et al.*, 2010 ▸), GUTZAX (Wang *et al.*, 2009 ▸), IPAGIQ (Wang *et al.*, 2011 ▸), TIVXED (Wang *et al.*, 2014*b* ▸), TIVXON (Wang *et al.*, 2014*b* ▸), UFETEF (Hu *et al.*, 2008 ▸), YATPAM (Liu, 2012 ▸), ZIPLOB (Chen *et al.*, 2010 ▸) *etc*]. In most cases, MEA behaves as a chelating ligand; however, there are metal complexes in which non-coordinating MEA mol­ecules are situated in the outer coordination sphere [AXUQAN (Ibragimov *et al.*, 2016*c* ▸), FAFTOV (Spitsin *et al.*, 1986 ▸), TIRQEQ (Halvorson *et al.*, 1995 ▸), WUZZOH (Guzei *et al.*, 2010*b* ▸) *etc*]. Mixed-ligand metal complexes including BTA and MEA have not been reported in the CSD up to date.

## Synthesis and crystallization   

To an aqueous solution (2.5 ml) of CuCl_2_·2H_2_O (0.048 g, 0.282 mmol) was slowly added an ethanol solution (5 ml) containing MEA (0.034 g, 0.565 mmol) and HBTA (0.1 g, 0.565 mmol) under constant stirring. Blue crystals of the product were obtained by solvent evaporation at room temperature after one week. Yield: 70%. Elemental analysis: Calc. for C_20_H_26_CuN_8_O_6_ (538.04): C, 44.65; H, 4.87 N, 20.83%. Found: C, 44.73; H, 4.93; N, 20.88%.

## Refinement   

Crystal data, data collection and structure refinement details are summarized in Table 2 ▸. The C-bound hydrogen atoms were placed in calculated positions and refined as riding atoms with C—H = 0.93 and 0.97 Å for aromatic and methyl­ene hydrogen atoms, respectively, and with *U*_iso_(H) = 1.2*U*_eq_(C). The positions of the O- and N bound H atoms were located from a difference-Fourier map and were refined with soft distance restraints, 0.82 Å for the hydroxyl group and 0.95 Å for the primary amine group.

## Supplementary Material

Crystal structure: contains datablock(s) I. DOI: 10.1107/S2056989019000744/wm5481sup1.cif

Structure factors: contains datablock(s) I. DOI: 10.1107/S2056989019000744/wm5481Isup2.hkl

CCDC reference: 1891272

Additional supporting information:  crystallographic information; 3D view; checkCIF report

## Figures and Tables

**Table 1:** Hydrogen-bond geometry (Å, °)

*D*—H⋯*A*	*D*—H	H⋯*A*	*D*⋯*A*	*D*—H⋯*A*
O3—H3⋯O1^i^	0.80 (1)	1.86 (1)	2.634 (2)	163 (3)
N4—H4*A*⋯O2^ii^	0.89 (1)	2.41 (2)	3.046 (2)	129 (2)
N4—H4*B*⋯O3^ii^	0.89 (1)	2.12 (1)	2.973 (2)	161 (2)
C7—H7*A*⋯O1^iii^	0.97	2.53	3.449 (3)	158
C9—H9*A*⋯N3^iv^	0.97	2.58	3.345 (3)	136

Symmetry codes: (i) 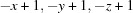; (ii) 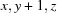; (iii) 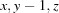; (iv) 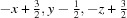.

**Table 2:** Experimental details

Crystal data	
Chemical formula	[Cu(C_8_H_6_N_3_O_2_)_2_(C_2_H_7_NO)_2_]
*M*_r_	538.03
Crystal system, space group	Monoclinic, *P*2_1_/*n*
Temperature (K)	293
*a*, *b*, *c* (Å)	12.4283 (4), 4.84866 (9), 20.6944 (5)
β (°)	105.823 (3)
*V* (Å^3^)	1199.80 (5)
*Z*	2
Radiation type	Cu *K*α
μ (mm^−1^)	1.75
Crystal size (mm)	0.36 × 0.22 × 0.12

Data collection	
Diffractometer	Rigaku Xcalibur Ruby
Absorption correction	Multi-scan (*CrysAlis PRO*; Rigaku OD, 2015 ▸)
*T*_min_, *T*_max_	0.558, 1.000
No. of measured, independent and observed [*I* > 2σ(*I*)] reflections	8833, 2444, 2152
*R*_int_	0.031
(sin θ/λ)_max_ (Å^−1^)	0.629

Refinement	
*R*[*F* ^2^ > 2σ(*F* ^2^)], *wR*(*F* ^2^), *S*	0.033, 0.096, 1.06
No. of reflections	2444
No. of parameters	173
No. of restraints	3
H-atom treatment	H atoms treated by a mixture of independent and constrained refinement
Δρ_max_, Δρ_min_ (e Å^−3^)	0.24, −0.31

Computer programs: *CrysAlis PRO* (Rigaku OD, 2015 ▸), *SHELXT* (Sheldrick, 2015*a* ▸), *SHELXL2014* (Sheldrick, 2015*b* ▸), *Mercury* (Macrae *et al.*, 2006 ▸ and *publCIF* (Westrip, 2010 ▸).

## supplementary crystallographic information

### Crystal data

**Table:**

[Cu(C_8_H_6_N_3_O_2_)_2_(C_2_H_7_NO)_2_]	*F*(000) = 558
*M**_r_* = 538.03	*D*_x_ = 1.489 Mg m^−^^3^
Monoclinic, *P*2_1_/*n*	Cu *K*α radiation, λ = 1.54184 Å
*a* = 12.4283 (4) Å	Cell parameters from 3723 reflections
*b* = 4.84866 (9) Å	θ = 3.7–75.7°
*c* = 20.6944 (5) Å	µ = 1.75 mm^−^^1^
β = 105.823 (3)°	*T* = 293 K
*V* = 1199.80 (5) Å^3^	Block, blue
*Z* = 2	0.36 × 0.22 × 0.12 mm

### Data collection

**Table:**

Rigaku Xcalibur Ruby diffractometer	2444 independent reflections
Radiation source: fine-focus sealed X-ray tube	2152 reflections with *I* > 2σ(*I*)
Detector resolution: 10.2576 pixels mm^-1^	*R*_int_ = 0.031
ω scans	θ_max_ = 75.9°, θ_min_ = 3.8°
Absorption correction: multi-scan (CrysAlis PRO; Rigaku OD, 2015)	*h* = −15→15
*T*_min_ = 0.558, *T*_max_ = 1.000	*k* = −5→5
8833 measured reflections	*l* = −20→25

### Refinement

**Table:**

Refinement on *F*^2^	Hydrogen site location: mixed
Least-squares matrix: full	H atoms treated by a mixture of independent and constrained refinement
*R*[*F*^2^ > 2σ(*F*^2^)] = 0.033	*w* = 1/[σ^2^(*F*_o_^2^) + (0.048*P*)^2^ + 0.364*P*] where *P* = (*F*_o_^2^ + 2*F*_c_^2^)/3
*wR*(*F*^2^) = 0.096	(Δ/σ)_max_ < 0.001
*S* = 1.06	Δρ_max_ = 0.24 e Å^−^^3^
2444 reflections	Δρ_min_ = −0.31 e Å^−^^3^
173 parameters	Extinction correction: SHELXL2014 (Sheldrick, 2015b), Fc^*^=kFc[1+0.001xFc^2^λ^3^/sin(2θ)]^-1/4^
3 restraints	Extinction coefficient: 0.0009 (2)

### Special details

**Table:**

Geometry. All esds (except the esd in the dihedral angle between two l.s. planes) are estimated using the full covariance matrix. The cell esds are taken into account individually in the estimation of esds in distances, angles and torsion angles; correlations between esds in cell parameters are only used when they are defined by crystal symmetry. An approximate (isotropic) treatment of cell esds is used for estimating esds involving l.s. planes.

### Fractional atomic coordinates and isotropic or equivalent isotropic displacement parameters (Å^2^)

**Table:**

	*x*	*y*	*z*	*U*_iso_*/*U*_eq_	
Cu	0.5000	0.5000	0.5000	0.03515 (14)	
O2	0.53163 (12)	0.2852 (3)	0.58733 (6)	0.0434 (3)	
O3	0.66203 (12)	0.2720 (3)	0.47288 (7)	0.0449 (3)	
O1	0.46322 (14)	0.5886 (3)	0.64711 (7)	0.0535 (4)	
N1	0.56782 (14)	0.3490 (4)	0.76528 (8)	0.0420 (4)	
N4	0.62260 (16)	0.7586 (3)	0.54159 (8)	0.0432 (4)	
N3	0.60695 (19)	0.6560 (5)	0.84410 (10)	0.0659 (6)	
C7	0.57879 (18)	0.2195 (5)	0.70430 (9)	0.0458 (5)	
H7A	0.5484	0.0342	0.7013	0.055*	
H7B	0.6574	0.2054	0.7061	0.055*	
N2	0.64071 (17)	0.5465 (5)	0.79544 (10)	0.0582 (5)	
C8	0.51797 (16)	0.3819 (4)	0.64159 (9)	0.0383 (4)	
C3	0.50966 (19)	0.5287 (5)	0.84514 (11)	0.0494 (5)	
C2	0.48288 (17)	0.3334 (4)	0.79457 (10)	0.0430 (4)	
C10	0.74518 (19)	0.4794 (5)	0.49439 (13)	0.0568 (6)	
H10A	0.7391	0.6123	0.4585	0.068*	
H10B	0.8187	0.3956	0.5041	0.068*	
C9	0.73262 (19)	0.6246 (5)	0.55560 (12)	0.0557 (5)	
H9A	0.7405	0.4930	0.5919	0.067*	
H9B	0.7910	0.7621	0.5696	0.067*	
C1	0.3878 (2)	0.1733 (5)	0.78222 (14)	0.0628 (6)	
H1	0.3704	0.0434	0.7479	0.075*	
C4	0.4385 (3)	0.5704 (7)	0.88710 (14)	0.0763 (8)	
H4	0.4549	0.7005	0.9214	0.092*	
C6	0.3200 (3)	0.2185 (7)	0.82423 (19)	0.0817 (9)	
H6	0.2551	0.1146	0.8180	0.098*	
C5	0.3452 (3)	0.4120 (7)	0.87504 (19)	0.0849 (10)	
H5	0.2967	0.4345	0.9018	0.102*	
H4A	0.609 (2)	0.836 (6)	0.5773 (10)	0.078 (9)*	
H4B	0.623 (2)	0.895 (4)	0.5130 (11)	0.063 (7)*	
H3	0.6329 (19)	0.295 (5)	0.4336 (6)	0.055 (7)*	

### Atomic displacement parameters (Å^2^)

**Table:**

	*U*^11^	*U*^22^	*U*^33^	*U*^12^	*U*^13^	*U*^23^
Cu	0.0477 (2)	0.0285 (2)	0.0312 (2)	0.00425 (15)	0.01401 (15)	0.00123 (13)
O2	0.0614 (9)	0.0372 (7)	0.0338 (6)	0.0079 (6)	0.0165 (6)	0.0036 (5)
O3	0.0546 (8)	0.0376 (7)	0.0420 (7)	0.0017 (6)	0.0123 (6)	0.0019 (6)
O1	0.0741 (10)	0.0485 (8)	0.0375 (7)	0.0204 (8)	0.0144 (7)	0.0023 (6)
N1	0.0441 (9)	0.0473 (10)	0.0336 (8)	−0.0002 (7)	0.0088 (6)	0.0049 (7)
N4	0.0584 (10)	0.0309 (9)	0.0406 (9)	0.0015 (7)	0.0143 (8)	−0.0008 (7)
N3	0.0681 (13)	0.0681 (14)	0.0559 (11)	−0.0137 (11)	0.0077 (10)	−0.0147 (10)
C7	0.0558 (12)	0.0469 (11)	0.0350 (9)	0.0103 (9)	0.0130 (8)	0.0053 (8)
N2	0.0507 (11)	0.0671 (13)	0.0535 (11)	−0.0122 (9)	0.0085 (8)	−0.0018 (9)
C8	0.0472 (10)	0.0361 (10)	0.0322 (8)	0.0001 (8)	0.0119 (7)	0.0025 (7)
C3	0.0562 (12)	0.0505 (13)	0.0401 (10)	0.0043 (10)	0.0109 (9)	0.0045 (9)
C2	0.0446 (10)	0.0441 (11)	0.0385 (9)	0.0040 (8)	0.0082 (8)	0.0103 (8)
C10	0.0449 (11)	0.0573 (14)	0.0721 (15)	−0.0031 (10)	0.0227 (11)	−0.0066 (11)
C9	0.0502 (12)	0.0472 (13)	0.0611 (13)	−0.0009 (10)	0.0009 (10)	−0.0049 (10)
C1	0.0580 (14)	0.0556 (15)	0.0722 (15)	−0.0095 (11)	0.0133 (12)	0.0092 (12)
C4	0.105 (2)	0.0715 (18)	0.0614 (15)	0.0215 (17)	0.0369 (16)	0.0050 (13)
C6	0.0641 (16)	0.0718 (19)	0.121 (3)	0.0015 (14)	0.0447 (17)	0.0307 (19)
C5	0.087 (2)	0.082 (2)	0.105 (2)	0.0210 (18)	0.060 (2)	0.0341 (19)

### Geometric parameters (Å, º)

**Table:**

Cu—N4^i^	1.9798 (18)	C7—H7A	0.9700
Cu—N4	1.9798 (18)	C7—H7B	0.9700
Cu—O2	2.0292 (12)	C3—C2	1.383 (3)
Cu—O2^i^	2.0293 (12)	C3—C4	1.413 (4)
Cu—O3^i^	2.4917 (15)	C2—C1	1.378 (3)
O2—C8	1.270 (2)	C10—C9	1.494 (3)
O3—C10	1.424 (3)	C10—H10A	0.9700
O3—H3	0.802 (10)	C10—H10B	0.9700
O1—C8	1.234 (2)	C9—H9A	0.9700
N1—N2	1.349 (3)	C9—H9B	0.9700
N1—C2	1.355 (3)	C1—C6	1.383 (4)
N1—C7	1.449 (2)	C1—H1	0.9300
N4—C9	1.470 (3)	C4—C5	1.356 (5)
N4—H4A	0.885 (10)	C4—H4	0.9300
N4—H4B	0.887 (10)	C6—C5	1.380 (5)
N3—N2	1.305 (3)	C6—H6	0.9300
N3—C3	1.363 (3)	C5—H5	0.9300
C7—C8	1.530 (3)		
			
N4^i^—Cu—N4	180.0	N3—C3—C4	130.7 (3)
N4^i^—Cu—O2	90.08 (6)	C2—C3—C4	120.1 (2)
N4—Cu—O2	89.92 (6)	N1—C2—C3	104.13 (18)
N4^i^—Cu—O2^i^	89.92 (6)	N1—C2—C1	132.9 (2)
N4—Cu—O2^i^	90.08 (6)	C3—C2—C1	122.9 (2)
O2—Cu—O2^i^	180.0	O3—C10—C9	111.30 (18)
C8—O2—Cu	123.91 (12)	O3—C10—H10A	109.4
C10—O3—H3	107.8 (19)	C9—C10—H10A	109.4
N2—N1—C2	109.84 (17)	O3—C10—H10B	109.4
N2—N1—C7	120.00 (17)	C9—C10—H10B	109.4
C2—N1—C7	129.44 (18)	H10A—C10—H10B	108.0
C9—N4—Cu	111.72 (13)	N4—C9—C10	110.35 (18)
C9—N4—H4A	113.6 (19)	N4—C9—H9A	109.6
Cu—N4—H4A	110 (2)	C10—C9—H9A	109.6
C9—N4—H4B	106.4 (17)	N4—C9—H9B	109.6
Cu—N4—H4B	109.1 (18)	C10—C9—H9B	109.6
H4A—N4—H4B	106 (3)	H9A—C9—H9B	108.1
N2—N3—C3	107.5 (2)	C2—C1—C6	115.6 (3)
N1—C7—C8	111.94 (16)	C2—C1—H1	122.2
N1—C7—H7A	109.2	C6—C1—H1	122.2
C8—C7—H7A	109.2	C5—C4—C3	116.9 (3)
N1—C7—H7B	109.2	C5—C4—H4	121.5
C8—C7—H7B	109.2	C3—C4—H4	121.5
H7A—C7—H7B	107.9	C5—C6—C1	122.4 (3)
N3—N2—N1	109.23 (18)	C5—C6—H6	118.8
O1—C8—O2	126.26 (17)	C1—C6—H6	118.8
O1—C8—C7	119.79 (16)	C4—C5—C6	122.0 (3)
O2—C8—C7	113.94 (17)	C4—C5—H5	119.0
N3—C3—C2	109.3 (2)	C6—C5—H5	119.0
			
N2—N1—C7—C8	−86.8 (2)	C7—N1—C2—C1	8.5 (4)
C2—N1—C7—C8	82.4 (3)	N3—C3—C2—N1	0.9 (2)
C3—N3—N2—N1	−0.6 (3)	C4—C3—C2—N1	−179.9 (2)
C2—N1—N2—N3	1.2 (2)	N3—C3—C2—C1	−178.9 (2)
C7—N1—N2—N3	172.31 (19)	C4—C3—C2—C1	0.3 (3)
Cu—O2—C8—O1	16.3 (3)	Cu—N4—C9—C10	50.9 (2)
Cu—O2—C8—C7	−162.68 (14)	O3—C10—C9—N4	−60.3 (3)
N1—C7—C8—O1	−3.5 (3)	N1—C2—C1—C6	179.8 (2)
N1—C7—C8—O2	175.61 (17)	C3—C2—C1—C6	−0.4 (3)
N2—N3—C3—C2	−0.2 (3)	N3—C3—C4—C5	179.0 (3)
N2—N3—C3—C4	−179.3 (3)	C2—C3—C4—C5	−0.1 (4)
N2—N1—C2—C3	−1.2 (2)	C2—C1—C6—C5	0.4 (4)
C7—N1—C2—C3	−171.29 (19)	C3—C4—C5—C6	0.0 (5)
N2—N1—C2—C1	178.6 (2)	C1—C6—C5—C4	−0.1 (5)

Symmetry code: (i) −*x*+1, −*y*+1, −*z*+1.

### Hydrogen-bond geometry (Å, º)

**Table:**

*D*—H···*A*	*D*—H	H···*A*	*D*···*A*	*D*—H···*A*
O3—H3···O1^i^	0.80 (1)	1.86 (1)	2.634 (2)	163 (3)
N4—H4*A*···O2^ii^	0.89 (1)	2.41 (2)	3.046 (2)	129 (2)
N4—H4*B*···O3^ii^	0.89 (1)	2.12 (1)	2.973 (2)	161 (2)
C7—H7*A*···O1^iii^	0.97	2.53	3.449 (3)	158
C9—H9*A*···N3^iv^	0.97	2.58	3.345 (3)	136

Symmetry codes: (i) −*x*+1, −*y*+1, −*z*+1; (ii) *x*, *y*+1, *z*; (iii) *x*, *y*−1, *z*; (iv) −*x*+3/2, *y*−1/2, −*z*+3/2.
